# Observational, prospective, multicentre study to evaluate the effects of counselling on the choice of combined hormonal contraceptives in Italy—the ECOS (Educational COunselling effectS) study

**DOI:** 10.1186/s12905-015-0226-x

**Published:** 2015-09-02

**Authors:** Alessandro Gambera, Fedela Corda, Rosetta Papa, Carlo Bastianelli, Sandra Bucciantini, Salvatore Dessole, Pasquale Scagliola, Nadia Bernardini, Daniela de Feo, Fabiola Beligotti

**Affiliations:** Spedali Civili di Brescia, Clinica Universitaria, Brescia, Italy; Centro Donna c/o Ospedale Binaghi, Cagliari, Italy; UOC Tutela Salute Donna- ASL Napoli 1 CENTRO, Naples, Italy; Policlinico Umberto I, Dipartimento di Ginecologia e Ostetricia, Rome, Italy; Azienda Universitaria Careggi, Divisione Clinica Ostetrica, Florence, Italy; A.O.U. di Sassari Macrostruttura Materno Infantile Clinica Ostetrico Ginecologica, Sassari, Italy; MSD Italia S.r.L., a subsidiary of Merck & Co., Inc., Whitehouse Station, NJ USA

## Abstract

**Background:**

Adequate counselling on contraceptive methods can help users choose the most appropriate method. The aim of this study was to assess the effects of structured counselling provided by gynaecologists on selection of a combined hormonal contraception method.

**Methods:**

Women aged 18–40 years (*n* = 1871) who were considering the use of a combined hormonal contraception method (pill, transdermal patch or vaginal ring) underwent a structured counselling session in which gynaecologists provided comprehensive information. Pre- and post-counselling questionnaires on combined hormonal contraception choice were completed by participants.

**Results:**

After counselling, many women (38 %) selected a combined hormonal contraception method that was different from the originally intended one. Preferences for the transdermal patch approximately doubled (from 3.2 % pre-counselling to 7 %; *p* < 0.0001) and those for the vaginal ring increased four-fold (from 5.2 to 21.2 %; *p* < 0.0001), while preference for the pill remained unchanged (from 64.5 % [pre-] to 64.1 % [post-counselling]). The proportion of undecided women decreased from 18 to 2.1 % (*p* < 0.0001). The main reasons for choosing a method were related to ease of use (all methods), and preferences for administration frequency (daily, weekly or monthly). The number of patients requiring post-counselling contact with the physician’s office was low (5.1–6.9 %), as was the incidence of adverse events (1.8–3.1 %).

**Conclusions:**

Counselling has a significant impact on women’s choice of combined hormonal contraception and encourages them to consider alternative methods to combined oral contraceptives. Moreover, it also enables women to use their chosen method with confidence.

**Trial registration:**

NCT01181778, Trial registration date: August 12, 2010

**Electronic supplementary material:**

The online version of this article (doi:10.1186/s12905-015-0226-x) contains supplementary material, which is available to authorized users.

## Background

Among the numerous contraceptive options available to women in Western countries, combined oral contraceptives (COCs; the daily ‘pill’) remain the most common and recommended form of reversible contraception, although with country-specific differences [1–6]. The range of combined hormonal contraceptive (CHC) options has increased with development of the transdermal patch and the vaginal ring. The efficacy and safety of these methods are similar to those of COCs, but they differ from the pill in the route of hormone delivery and frequency of administration (weekly for the transdermal patch and monthly for the vaginal ring) [7, 8]. Hormone released the transdermal patch or the vaginal ring is independent of gastrointestinal absorption, while transvaginal absorption—when using the ring—also provides a substantial reduction of the daily fluctuations in steroid levels observed with COCs, allowing the use of lower doses of ethinylestradiol [9, 10].

However, women’s knowledge of newer contraceptive methods is still limited. Indeed, the wide range of contraceptive options available today makes the process of selection an increasingly difficult task. Many factors also affect the choice of a contraceptive: efficacy in preventing pregnancy and safety/tolerability are obviously important, but so are ease of use, convenience, effects on menstrual bleeding, benefits or risks for general health (true or perceived), as well as partners’ or peers’ attitudes [3, 4, 11, 12]. Sociocultural and personal factors also play an important role in the choice of a birth control method [6, 13].

Adequate information from healthcare professionals (HCPs), through counselling, is an essential step in helping women to select the contraceptive method that best suits their individual needs [14]. Counselling may clarify doubts or erroneous perceptions, and allow counsellors to suggest the best method for a woman’s clinical profile and lifestyle, and encourage an open dialogue about sexual well-being. Counselling may also anticipate common concerns that may arise after starting a particular method, thus setting realistic expectations about possible adverse events (AEs) and reducing the need for post-counselling contact with physician offices (callbacks).

Recently, the Contraceptive Health Research of Informed Choice Experience (CHOICE) study assessed the effect of structured counselling on personal contraceptive decisions, and explored the reasons for these choices. In that study, counselling increased the use of alternative CHCs, though there were substantial differences between countries. The CHOICE study was conducted in a group of 11 European countries that did not include Italy.

Italy has one of the lowest rates of COC use in Europe (15.2 %) [1], and the aim of the present study, the Educational COunselling effectS (ECOS) study, was to assess the effects of structured counselling provided by gynaecologists on selection of a CHC method (pill, transdermal patch or vaginal ring) by women from Italy.

## Methods

### Design

This observational, prospective study was conducted in 16 gynaecology centres (in hospitals or public health centres throughout Italy, see Additional file 1) between November 11, 2010 and October 30, 2012. The objective of the study was to monitor the effects of counselling on combined hormonal contraceptive choice using a standardised counselling questionnaire (see Additional file 2) and guide. The study was conducted in accordance with European regulations regarding non-interventional studies, and the protocol was reviewed and approved by independent ethics committees for all centres. The study consisted of a screening phase and an observational phase. The screening phase followed methodology established in the European CHOICE study [15]. During the observational phase, physicians recorded all callbacks and the reasons for calling back.

### Participants

Healthy women aged ≥18 and ≤40 years who were considering starting a pill, transdermal patch or vaginal ring CHC method (or re-starting one after a ≥1-month suspension), who were not interested in initiating a pregnancy in the next 3 months and who agreed to complete a questionnaire were invited to participate in the study. Women who consulted their physician to stop a CHC method could not participate (unless they intended to switch from one COC to another method). Women who requested a CHC method but for whom the gynaecologist considered another method more appropriate (e.g., presence of contraindications for CHC) were appropriately counselled but still completed the questionnaire. Women were asked to sign an informed consent form prior to enrolment.

### Observational measures

At the start of the observation phase, physicians compiled part A of the questionnaire with information on which method the participant intended to use (if any), as well as whether CHC was indicated for this participant and whether the counselling leaflet was used (see Additional file 3).

The counselling leaflet (an Italian translation of the one used in the European CHOICE study [15] which had been developed in consultation with the European Society of Contraception and Reproductive Health) [12], and presented a comprehensive and balanced overview of the three CHC methods, including reference to alternative progestogen-only methods. Counselling provided concise information on mode of action, frequency of administration, proper use, efficacy, tolerability, potential risks and benefits, suitability for individual needs and other features that the gynaecologist might consider important for the individual woman.

Subsequently, the subjects compiled part B of the questionnaire, in which they provided demographic information, including age, educational level, employment status, number of children, wish to have children in the future, history of unplanned pregnancies or abortions, the presence of a partner and the most recently used contraceptive method. They indicated which method (if any) they had chosen, the reasons for selecting that method and the reasons for not choosing the other contraceptive options. They then provided their perceptions about the three CHC methods (pill, transdermal patch or vaginal ring). Perceptions were investigated by means of multiple-choice statements (strongly agree, agree, no opinion, disagree, strongly disagree, do not know) to the following eight statements about each method: The method prevents pregnancy effectively; has many side effects; can be dangerous for your health; is easy to use; is easy to forget; gives you regular menstrual bleeding; protects against certain forms of cancer; many women use the method.

The study also included a 4-month follow-up, during which data on the number of users who contacted the physician’s office to report concerns, problems or adverse events with the chosen contraceptive method (i.e., callbacks) were collected. The reasons for calling were grouped in four major categories: side effects, non-compliant behaviour, doubts or fears, and other reasons. Low callback rates were considered an indicator of both satisfaction and counselling clarity; moreover, it was hypothesized that callback rates might differ depending on the chosen method.

All AEs reported during the 4-month follow-up were collected according to regulations on spontaneous reporting in a postmarketing setting and reported on the case report form, specifying the duration and severity of each event, and its relationship to the study treatment. The study was approved by the Ethics Committee of Azienda Universitaria Careggi, Divisione Clinica Ostetrica, Florence, Italy.

### Statistical analyses

Statistical analyses were performed using descriptive methods. Absolute frequency distributions of hormonal contraceptive method chosen by patients before and after counselling (the principal endpoint of the study) are presented. Difference in distribution of CHC methods before and after counselling was tested with McNemar’s chi-squared tests and presented with two-sided 95 % confidence intervals (CIs). The differences in proportions for the pre- versus post-counselling choice were assessed at a one-sided significance level of 1.25 % (significance adjusted for multiple comparisons). Descriptive statistics on all characterisation variables were provided overall and for each post-counselling CHC group. For the exploratory analyses a two-sided significance level of 5 % was used. With regard to callbacks, patients who called back more than once and those having multiple complaints were counted overall and for each method.

Demographic and social variables were evaluated (e.g., age, weight, place of residence, marital status, education, employment status, number of children, planning of future pregnancies, unintended pregnancies, abortions and stability of relationship with partner).

Based on data on contraceptive use in Italy, where approximately 20 % of women consulting their physicians would be interested in a CHC method [6] (and considering an 80–90 % prevalence of COC reported in the 2010 IMS Health Report), a sample size of 1850 women was deemed sufficient to detect a difference post- versus pre-counselling for ring or patch of 3 % with an overall power of 90 %. This sample size also permitted assessment of a 10 % callback rate reduction in the vaginal ring group versus the pill group as significant (alpha = 0.05, power = 80 %). All analyses were performed with the SAS (version 8) statistical software package.

## Results

### Participants

A total of 1919 women were included in the observational phase. Their characteristics are summarised in Table 1. Before the consultation, the most frequently used contraceptive method was the pill (36.8 %), followed by condoms (28.3 %), while 18.2 % of women had never previously ever used contraceptive methods. The transdermal patch and the vaginal ring had been used, respectively, by 1.8 and 2.7 % of women. Physicians did not consider CHC to be a suitable method for 48 (2.5 %) participating women, mainly due to the presence of contraindications or medical conditions. In all, 1871 (97.5 %) were included in the analyses.

**Table 1:** Baseline characteristics of the study population

Parameter^a^	Participants (*n* = 1919)
Age, years, mean (SD)	26.6 (6.3)
Weight, kg^b^, mean (SD)	58.6 (19.0)
Residence, *n* (%)	
Urban	1559 (81.2)
Rural	320 (16.7)
Marital status, *n* (%)	
Never married	1430 (74.5)
Married	365 (19.0)
Separated/divorced	41 (2.1)
Education, *n* (%)	
None	2 (0.1)
Primary school	60 (3.1)
Secondary school	432 (22.5)
High school graduate	987 (51.4)
Bachelor’s degree	327 (17.0)
Employment status, *n* (%)	
None	95 (5.0)
Part-time employment	153 (8.0)
Full-time employment	589 (30.7)
Student	651 (33.9)
Housewife	178 (9.3)
Unemployed	220 (11.5)
Children, *n* (%)^b^	
0	1408 (73.4)
1	243 (12.7)
2	216 (11.3)
3	37 (1.9)
≥4	9 (0.5)
Planning future pregnancies, *n* (%)^b^	
Yes	1367 (71.5)
No	271 (14.2)
Do not know yet	260 (13.6)
Unplanned pregnancies, *n* (%)^b^	
0	1492 (78.0)
1	341 (17.8)
2	42 (2.2)
3	6 (0.3)
4	4 (0.2)
5	1 (0.1)
Induced abortions, *n* (%)^c^	
0	1200 (80.1)
1	187 (12.5)
2	29 (1.9)
3	3 (0.2)
4	4 (0.3)
Stable relationship with partner, *n* (%)^b^	
Yes	1555 (81.3)
No	346 (18.1)
Last contraceptive method use, *n* (%)^b^	
Combined oral contraceptive pill	704 (36.8)
Condoms	542 (28.3)
Natural family planning	153 (8.0)
Vaginal ring	52 (2.7)
Transdermal patch	35 (1.8)
Copper-releasing intrauterine device	29 (1.5)
Progestogen-only pill	23 (1.2)
Levonorgestrel-releasing intrauterine system	13 (0.7)
Injectable	6 (0.3)
Contraceptive implant	2 (0.1)
No method	348 (18.2)

^a^Some variables were missing or not reported

^b^1913 subjects analysed

^c^1498 subjects analysed

### Contraceptive choice prior to counselling

Before the counselling session, most women were oriented toward using the pill (64.5 %), while 18.0 % were undecided. Intentions to use the transdermal patch and the vaginal ring were expressed by 3.2 and 5.2 %, respectively (Table 2).

**Table 2:** Comparison of intended (pre-counselling) versus chosen (post-counselling) contraceptive method

Method	Pre-counselling	Post-counselling	Difference	*p*-value (McNemar’s test)
	*n* (%)	*n* (%)	(95 % CI)	
Pill	1207 (64.5)	1199 (64.1)	−0.4 (−2.6 to 1.8)	0.71
Transdermal patch	60 (3.2)	131 (7.0)	3.8 (2.6 to 5.0)	<0.0001
Vaginal ring	97 (5.2)	397 (21.2)	16.0 (14.3 to 17.8)	<0.0001
Other method	171 (9.1)	104 (5.6)	−3.6 (−5.0 to −2.2)	<0.0001
Undecided	336 (18.0)	40 (2.1)	−15.8 (−17.6 to 14.1)	<0.0001

### Contraceptive choice post-counselling

After counselling, the pill was chosen as contraceptive method by 64.1 % of women, the transdermal patch by 7.0 % and the vaginal ring by 21.2 % (Table 3). Non-CHC methods were chosen by 5.6 % of subjects, while 2.1 % remained undecided. Compared with the intentions manifested before counselling, the proportion of women choosing the pill remained practically unchanged, while preferences for the other methods increased significantly (*p* < 0.0001), by approximately two-fold for the transdermal patch and by four-fold for the vaginal ring. Counselling also significantly decreased the percentage of women who originally intended to use other methods or were undecided (*p* < 0.0001).

**Table 3:** Women’s choice of CHC method according to intended (pre-counselling) method (unchanged preferences underlined)

Method intended (pre-counselling)	Method chosen (post-counselling)				
	Pill	Patch	Ring	Other method	Undecided
	*n* (%)	*n* (%)	*n* (%)	*n* (%)	*n* (%)
Pill (**64.5**)^a^	980 (81.2)	34 (2.8)	166 (13.8)	21 (1.7)	6 (0.5)
Transdermal patch (**3.2**)^a^	14 (23.3)	29 (48.3)	16 (26.7)	1 (1.7)	0
Vaginal ring (**5.2**)^a^	4 (4.1)	5 (5.2)	85 (87.6)	2 (2.1)	1 (1.0)
Other method (**9.1**)^a^	58 (33.9)	11 (6.4)	48 (28.1)	48 (28.1)	6 (3.5)
Undecided (**18.0**)^a^	143 (42.6)	52 (15.5)	82 (24.4)	32 (9.5)	27 (8.0)
	1199 (**64.1**)^a^	131 (**7.0**)^a^	397 (**21.2**)^a^	104 (**5.6**)^a^	40 (**2.1**)^a^

^a^Pre- and post-counselling total percentages are in bold

Among women who originally intended to use the pill, 81.2 % actually chose the pill, whereas 13.8 % opted for the vaginal ring and 2.8 % for the transdermal patch (Table 3). Among women who originally intended to use the transdermal patch, 48.3 % actually chose this, while the other half opted for the pill (23.3 %) or vaginal ring (26.7 %). Most women who were initially oriented to use the vaginal ring actually selected this method (87.6 %), with only a small percentage opting for the pill (4.1 %) or the transdermal patch (5.2 %). In the group of women who were initially undecided, most were able to make a choice after counselling: 42.6 % opted for the pill, 24.4 % for the vaginal ring and 15.5 % for the transdermal patch.

**Fig. 1:**
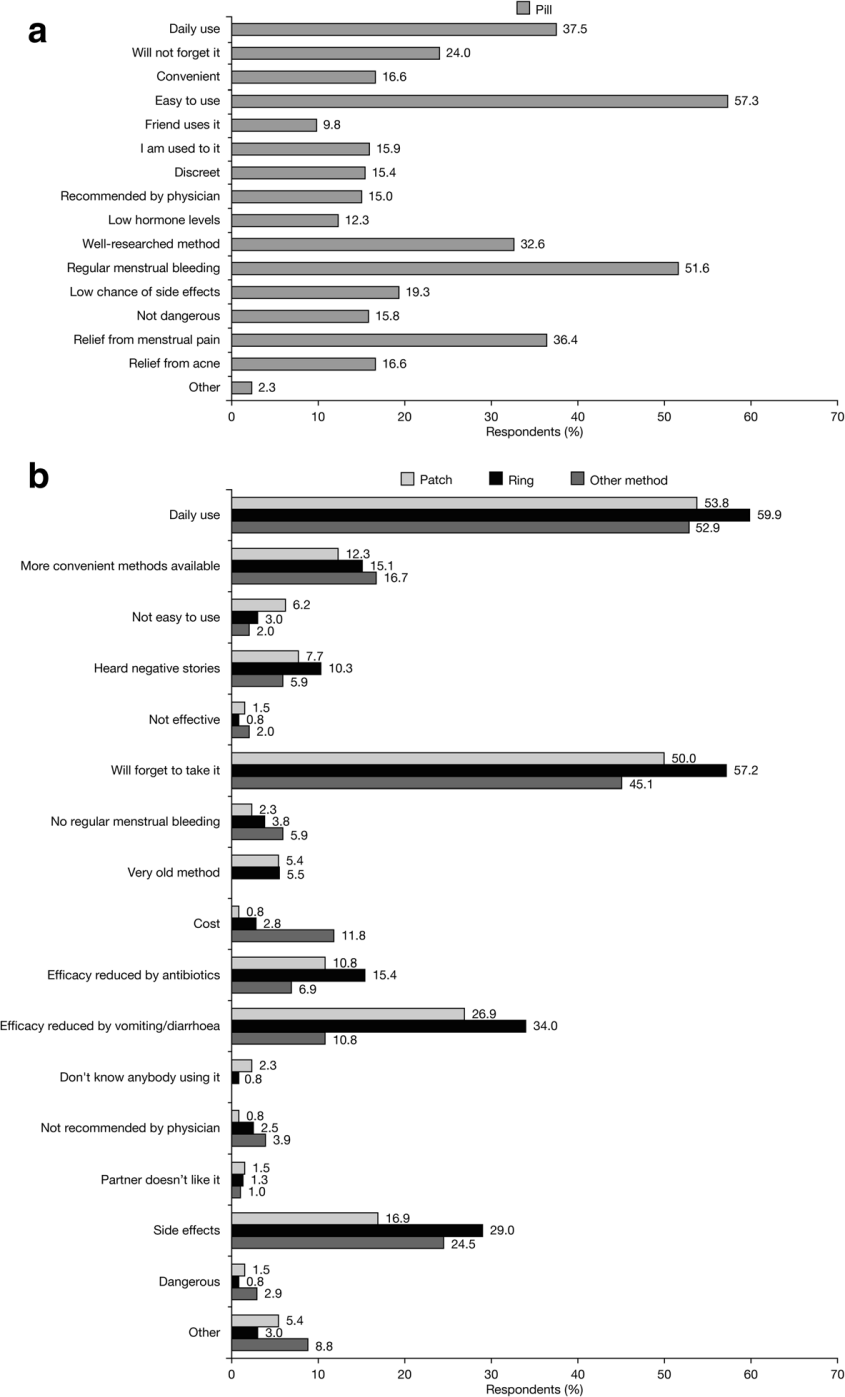
Main reasons for choosing the pill (**a**) and reasons for NOT choosing the pill versus method chosen (**b**) (percent of women stating each reason)

**Fig. 2:**
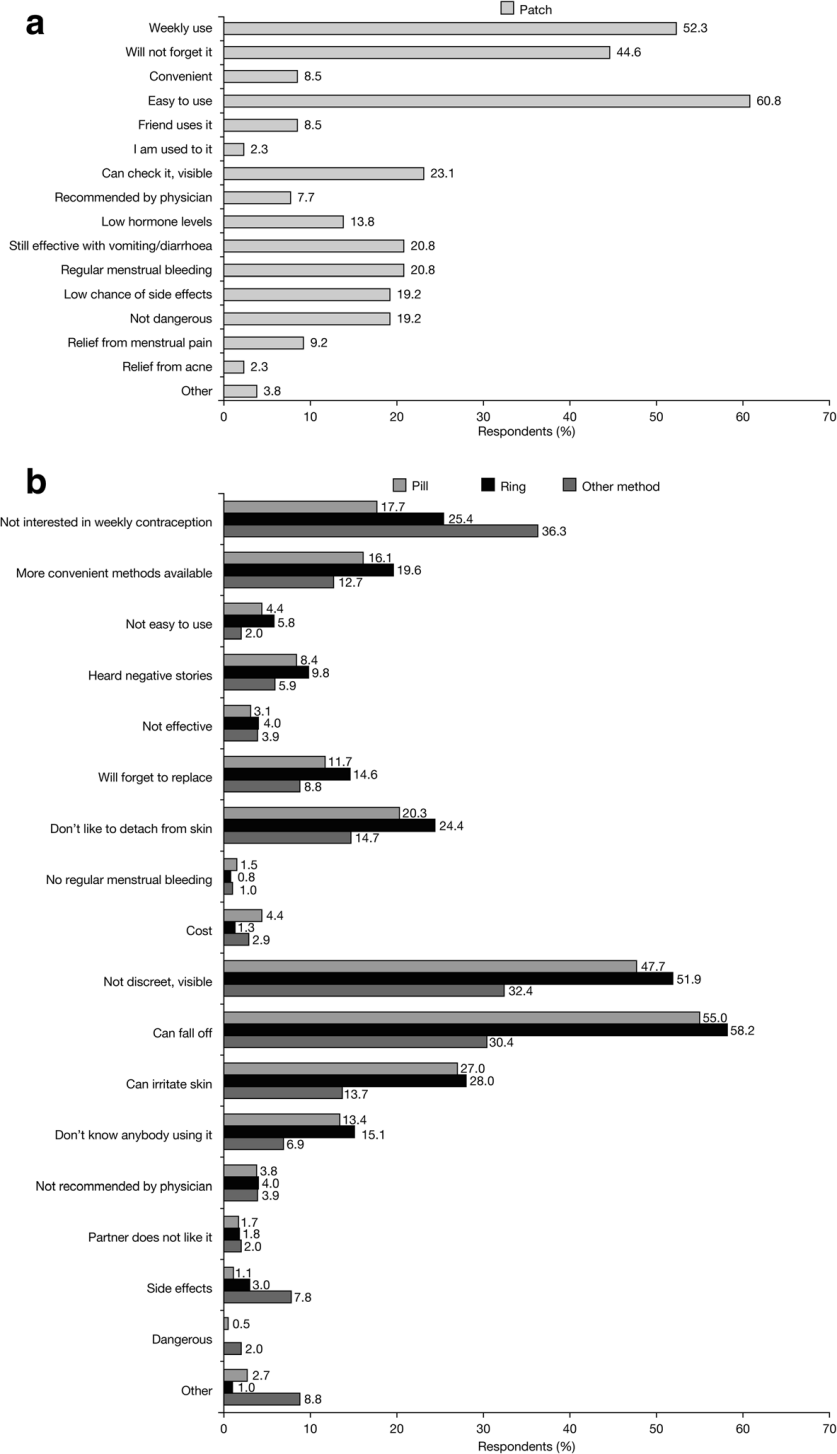
Main reasons for choosing the patch (**a**) and reasons for NOT choosing the patch versus method chosen (**b**) (percent of women stating each reason)

**Fig. 3:**
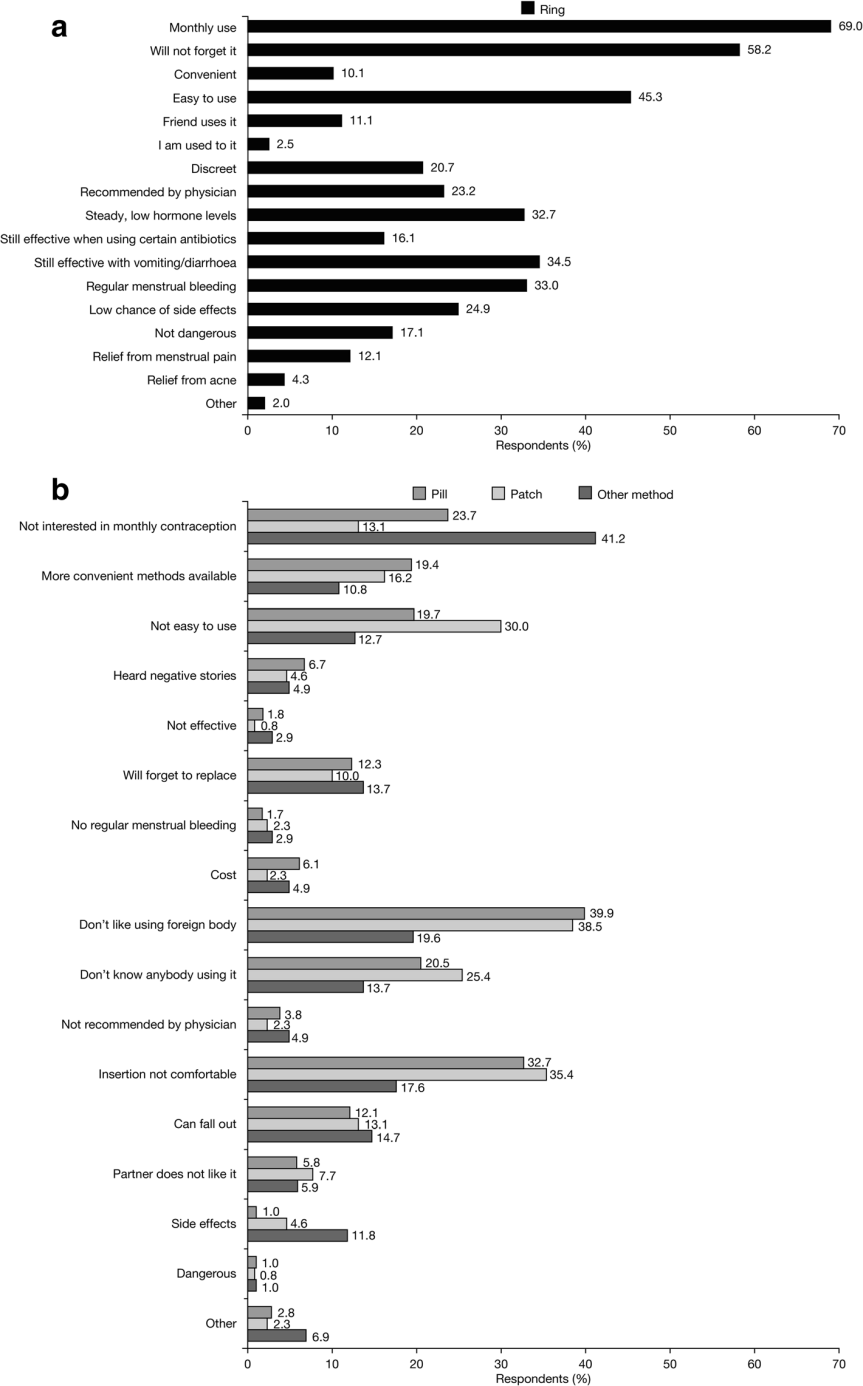
Main reasons for choosing the ring (**a**) and reasons for NOT choosing the ring versus method chosen (**b**) (percent of women stating each reason)

The reasons for choosing/not choosing the pill, the patch and the ring are summarised in Figs. 1, 2 and 3, respectively.

The information received during counselling was rated as very useful, complete and fair/balanced by 67.2, 63.0 and 62.4 % of participants, respectively, while 29.2, 33.0 and 31.4 %, respectively, rated it as sufficiently useful, complete and fair/balanced. The counselling leaflet was used in 85.6 % of all consultations; specifically, it was not used for users who requested a switch from one COC to another COC. Users were asked to rate the usefulness, completeness, and fairness of the information provided using a scale (very, sufficiently, neutral, not very, not at all). A high proportion of users had positive responses: very or sufficiently useful (96.4 %), very or sufficiently complete (96.0 %), and very or sufficiently fair/balanced (93.8 %).

### Effect of baseline characteristics as predictors for post-counselling contraceptive choice

No predictors of post-counselling contraceptive choice could be identified. The four groups (daily pill, transdermal patch, vaginal ring and other methods) did not differ with regard to age, weight and place of residence (rural vs urban). Unmarried women, who represented nearly 75 % of the study population, preferred the pill (with 80.8 % of subjects choosing the pill being unmarried) and the ring (72.5 %), with similar rates of preferences for the patch (63.4 %) or a method yet to be decided (67.5 %), while married women preferred other methods (42.3 %). The majority of women with a high school diploma or bachelor’s degree preferred the vaginal ring (50.6 and 23.7 %, respectively) or the daily pill (55.0 and 16.0 %, respectively). Students preferred the pill (40.4 %) or the ring (31.5 %). The majority of women who preferred the pill or the ring had never experienced an unplanned pregnancy (84.3 and 74.2 %, respectively) or an abortion (86.3 and 74.9 %, respectively). The stability/instability of the relationship with a partner did not appear to influence the choice of contraceptive method.

### Follow-up

During the 4-month follow-up period, the rate of callbacks for each method was calculated. Overall, there was a low rate of callbacks for any reason, with no significant differences among groups. The proportion of women calling their physician’s office about side effects was 2.2 % for pill users, 1.0 % for vaginal ring users and 3.1 % for transdermal patch users, while calls for non-compliant behaviour (missing/delayed doses of pill or incorrect application/insertion/removal of ring or patch) were made by 1.1 % of pill users, 1.8 % of vaginal ring users and 0 % of transdermal patch users (Table 4). Only the rate of callbacks related to doubts/fears differed significantly among groups, being highest among women who had chosen the transdermal patch (3.1 %, vs 0.7 % of pill users and 2.5 % of vaginal ring users; *p* < 0.05).

**Table 4:** Reasons for callbacks to the gynaecologists’ office during the 4-month follow-up

Callback reason	Pill	Patch	Ring	*p*-value
	(*n* = 1199)	(*n* = 131)	(*n* = 397)	(Chi-square test)
	*n* (%)	*n* (%)	*n* (%)	
All reasons^a^	61 (5.1)	9 (6.9)	24 (6.0)	0.66
Doubts, fears	8 (0.7)	4 (3.1)	10 (2.5)	<0.05
Non-compliant behaviour^b^	13 (1.1)	0	7 (1.8)	0.24
Other reasons	16 (1.3)	1 (0.8)	3 (0.8)	0.59
Side effects	26 (2.2)	4 (3.1)	4 (1.0)	0.23

^a^Patients were counted once within ‘All reasons’ and for each specific reason

^b^Missing/delays in taking pill or application/insertion or removal of transdermal patch/vaginal ring

### Safety evaluation

The incidence of AEs was very low in all contraceptive groups and the most frequent organ system affected was the reproductive system (Table 5). Women who chose the vaginal ring experienced the lowest rate of AEs (1.8 %, compared with 2.3 % for pill users and 3.1 % for transdermal patch users). No severe AEs were reported; 35 were graded as mild and 10 as moderate. There was a similar distribution of mild and moderate AEs among study groups.

**Table 5:** Adverse events reported during 4-month follow-up by system organ class

System organ class/preferred term	Pill		Patch		Ring	
	(*n* = 1199)		(*n* = 131)		(*n* = 397)	
	*n* (%)	Events	*n* (%)	Events	*n* (%)	Events
		*n*		*n*		*n*
All	28 (2.3)	30	4 (3.1)	5	7 (1.8)	10
Cardiac disorders	0	0	0	0	1 (0.3)	1
Gastrointestinal disorders	2 (0.2)	2	0	0	1 (0.3)	1
Infections/infestations	0	0	0	0	1 (0.3)	1
Investigations	4 (0.3)	4	0	0	0	0
Metabolism and nutrition disorders	1 (0.1)	1	0	0	0	0
Nervous system disorders	9 (0.8)	9	1 (0.8)	1	2 (0.5)	3
Psychiatric disorders	1 (0.1)	1	0	0	1 (0.3)	1
Reproductive system and breast disorders	12 (1.0)	13	4 (3.1)	4	3 (0.8)	3

## Discussion

This is the largest study exploring women’s preferences and perceptions about different CHC methods in Italy since the multinational survey by Skouby et al. published in 2004 [6]. Preferences for the pill remained substantially unchanged in our study (from 64.5 % pre- to 64.1 % post-counselling), while the percentages of women who were oriented to use the transdermal patch (3.2 %)or the vaginal ring (5.2 %) were low initially but increased significantly post-counselling by approximately two-fold for the transdermal patch (7.0 %) and four-fold for the vaginal ring (21.2 %). Similarly, counselling significantly reduced the proportion of women who initially intended to use a non-CHC method (from 9.1 to 5.6 %) or were undecided (from 18.0 to 2.1 %). Preferences for the vaginal ring remained unchanged post-counselling in 87.6 % of women (compared with 81.2 % for the pill and 48.3 % for the transdermal patch), while a shift in favour of the vaginal ring was registered in 13.8 and 26.7 % of intended pill and transdermal patch users, respectively. Overall, 38 % of the women selected a CHC method that was different from the one they initially intended to use.

These data reflect the findings of recent studies conducted on CHC choice. In the largest of these investigations, the Contraceptive Health Research of Informed Choice Experience (European CHOICE) study, which collected data from more than 18,000 women in 11 countries (not including Italy), after counselling, 47 % of participants selected a CHC method different from the one originally planned, with significant increases in transdermal patch use from 5 to 8 %, and in vaginal ring use from 8 to 30 % (*p* < 0.0001 for both methods) [14]. Great differences were noted among participating countries, although an increase in preferences for the transdermal patch or the vaginal ring was observed in all countries. This trend was also observed in a Brazilian study, where counselling induced an even more marked shift from COC (from 66.5 to 53.7 %) in favour of other methods (from 8.9 to 14 % for the transdermal patch and from 6.7 to 16 % for the vaginal ring) [16].

In our study, the participants rated the counselling experience positively, with >93 % of women considering the quality of the information received to be either *very* or *adequately* useful, complete, and fair and balanced. The importance of individualised counselling for selecting an appropriate contraceptive method has been highlighted in numerous studies. In one multinational survey designed to assess attitudes and preferences of a large population of CHC users, knowledge about the range of available contraceptive methods was often limited, even in women already using CHC, and 53–73 % expressed an interest in learning more about alternative contraceptive delivery methods in general [3]. Despite the increasing availability of the Internet and the recognised role of media and peers as sources of information, HCPs (mostly gynaecologists in Italy) maintain a primary role in guiding women in the process of decision-making about contraceptives, as documented in recent studies [4, 14]. In our study, counselling empowered undecided women to make a choice and encouraged the participants to consider alternative CHC methods, although preferences for the pill remained substantially unchanged. The biggest change from pre-counselling intentions was the increase in preferences for the vaginal ring, as this was the final choice for a substantial number of women who had initially intended to use another method (Table 3).

When the reasons for selecting a particular method were analysed, prominent reasons (i.e., indicated by >30 % of subjects) for choosing the pill were found to be ease of use, possibility of regular menstrual bleeding, daily use, relief from menstrual pain and being a well-researched method. The main reasons in favour of the transdermal patch were ease of use, weekly use and low chance of forgetting to apply it, while those for choosing the vaginal ring were monthly use, low chance of forgetting to insert it, ease of use, steady/low hormonal levels, retained effectiveness in case of vomiting or diarrhoea and possibility of regular menstrual bleeding. These findings are in agreement with findings from similar investigations, in particular an analysis of the CHOICE study revealed that the perceived ease of use of a method (including preferences in dosing frequency) is often more important than considerations strictly related to efficacy, tolerability and general health benefits/risks [11]. However, it should be noted that in our study approximately one-third of women who chose the vaginal ring after counselling cited other benefits offered by this method: steady and low hormone dosage, effectiveness irrespective of vomiting/diarrhoea, and regular menstrual bleeding. Likewise, regular menstrual bleeding was an important motivation for more than half of the women choosing the pill.

The reasons for not choosing a particular method also provide important insights into contraceptive selection criteria. As expected, the main reasons for not selecting the pill (vs the method chosen) were daily administration and high chance of forgetting to take it. The main reasons against choosing the transdermal patch were its visibility and the possibility of it falling off, while the main reasons not to select the vaginal ring were dislike of the insertion of a foreign body and feeling uncomfortable with vaginal insertion. While some of the reasons indicated for not choosing a method are intrinsic characteristics of that method (e.g., dosing frequency), negative perceptions about the vaginal ring are subjective feelings that could be modified by counselling. Topics related to sexuality and vaginal manipulation are notoriously difficult to discuss and require well-developed communication skills from information providers, as most women are uncomfortable talking about these issues with their HCPs. However, an open dialogue about vaginal anatomy and functions could greatly improve women’s body knowledge and help them take full advantage of all available contraceptive options [17].

A unique feature of our study was the inclusion of a 4-month follow-up period during which callbacks to the gynaecologist’s office and the reasons motivating them were recorded. Callback rates have been used to gauge user satisfaction with CHC [18]. Overall, the rate of callbacks was low (≤6 %), suggesting that counselling may have explained how to deal with potential problems and anticipated concerns that were likely to arise during initial use. However, about one third of women had used some form of CHC previously and this might have contributed to the low rate. The small number of callbacks and relatively small number of patch and ring users precludes meaningful comparison among the methods.

All methods appeared to be well-tolerated during the follow-up period, with a percentage of women contacting their physicians for AEs ranging from 1 % among vaginal ring users to 3 % among transdermal patch users. The only significant difference among contraceptive groups was a higher rate of callbacks regarding doubts or fears among transdermal patch users. The tolerability of the three contraceptive options was confirmed by the low incidence of reported AEs and the lack of severe AEs.

This study has limitations that should be acknowledged, some intrinsic to the study design. Like many similar investigations, we included only women who were interested in starting or re-starting CHC. Women who were oriented toward other forms of contraception were excluded *a priori* from the investigation, as were very young women or those aged ≥40 years. These results, therefore, cannot be generalised to the whole population of women seeking advice about contraception in Italy. Also, we did not investigate background characteristics of the participating gynaecologists, such as personal contraceptive preferences (which are known to influence recommendations) [19], nor did we compare each woman’s choice with the method recommended or suggested (if any) by her gynaecologist. The opinions of HCPs have a profound impact on contraceptive decisions of women they counsel [14]. Therefore, we cannot, rule out a bias toward recommending alternatives to COCs among participating gynaecologists. However, most women rated the quality of the information provided during counselling as fair and balanced. Furthermore, a relatively low percentage of women (8–23 %) indicated physician’s recommendation as a reason for choosing a contraceptive. Finally, the 4-month follow-up period included in this study was inadequate for a complete assessment of satisfaction, tolerability and compliance with the chosen method. However, these were not the study aims. Problems related to the use of a new contraceptive are more frequent during the initial phase and are expected to decline with time. The observation period was sufficient to judge whether counselling had made women more comfortable with their chosen CHC method and to record doubts or concerns and AEs experienced during this initial period.

## Conclusions

Our findings provide further evidence that well-structured and balanced counselling can influence the choice of CHC. When adequately informed, a substantial number of women will select a CHC option that is different from the originally intended one, and that this choice is often in favour of alternatives to COCs. HCPs should be proactive when counselling on the benefits and risks associated with the use of hormonal contraceptives, and should also consider what women think about the effects of CHCs on their health and wellbeing.

Counselling encourages appropriate use and adherence, and may prevent unplanned pregnancies that result from inconsistent or incorrect use of contraceptives. The pill, the transdermal patch, and the vaginal ring are the currently available and commonly used combined hormonal contraceptives (CHCs). Although all are effective when used properly, incorrect and irregular use contributes greatly to their failure. The consequent unintended pregnancies may be preventable though effective counselling about contraception.

## Additional files

Additional file 1:
**Partecipating centers.** The file includes the complete list of the participating centers, together with the name of the principal investigator. (XLS 100 kb) Additional file 2:
**Questionnaire.** The file provides the questionnaire compiled by the subjects. (DOC 193 kb) Additional file 3:
**Leaflet.** The file provides the counselling leaflet distributed to the subjects. (PDF 2810 kb)

## Abbreviations

AE: Adverse events
CHC: Combined hormonal contraceptive
CI: Confidence intervals
COC: Combined oral contraceptive
HCP: Healthcare professional
