# Supplementary material for: Observational, prospective, multicentre study to evaluate the effects of counselling on the choice of combined hormonal contraceptives in Italy—the ECOS (Educational COunselling effectS) study
Source: BMC Womens Health. 2015 Sep 2;15:69. doi: 10.1186/s12905-015-0226-x (PMC4557636; doi:10.1186/s12905-015-0226-x)
Supplement: Additional file 2: — Questionnaire. The file provides the questionnaire compiled by the subjects. (DOC 193 kb) [file 12905_2015_226_MOESM2_ESM.doc]

**QUESTIONNAIRE**

**PART A – FOR THE PHYSICIAN**

Please answer these questions for the woman you have invited to participate in the Study.

1. **Prior to any contraceptive counseling, which method of contraception did the woman think she may want to use (please tick one answer):**

1 Combined contraceptive pill

2 Contraceptive transdermal patch

3 Contraceptive vaginal ring

4 Other method

5 No preconceived idea **If she had no preference, did you – prior to the counseling – think**

**a particular method was best for her?**

1 No preconceived preference

2 Combined contraceptive pill

3 Contraceptive transdermal patch

4 Contraceptive vaginal ring

5 Other method

1. **Is there a particular reason why none of the three combined hormonal contraceptive methods are suitable for this woman?**

1 Yes, there are particular reasons why none of the three combined hormonal methods are suitable:

1 Contraindications

2 Problems in past 🡪 please check if you can use counseling leaflet

3 Medical conditions 🡪 but do ask woman to complete questionnaire

4 Other

0 No, she can use a combined hormonal contraceptive methods (pill, patch or ring)

🡪 please use counseling leaflet

🡪 please ask woman to complete questionnaire

1. **Did you use the Counseling Leaflet for this woman?**

1 Yes

0 No

**PART B – FOR PARTICIPATING WOMEN**

Now that you have received information from your doctor about available contraceptive methods, please complete the rest of this questionnaire.

1. **Date of completion: ____//____ // ________ (Day/Month/Year)**
2. **Your age:**
3. **Highest educational level (Please tick only one answer):**

1 Primary school

2 Completed high school

3 College, advanced education after high school

4 University

1. **Employment status:**

1 Not employed

2 Parttime employment

3 Fulltime employed

1. **Number of children:**
2. **Do you plan to have (more) children later?**

1 Yes

0 No

3 Do not know yet

1. **Have you had unplanned pregnancies?**

1 Yes How many?

0 No

1. **Optional question:**

**Have you had induced abortions?**

1 Yes How many?

0 No

1. **Are you in a steady relationship with a partner?**

1 Yes

0 No

**10. Which method of contraception was your last contraceptive method (or current method, if you are still using a method)?** (main method only!)

1 Combined Oral Contraceptive Pill 7 Intra-uterine device (other than Mirena)

2 Estrogen-free / Progestogen-only Contraceptive Pill 8 Contraceptive implant (Implanon or like)

3 Contraceptive transdermal patch 9 Injection (Depo-Provera or like)

4 Contraceptive vaginal ring 10 Condoms

5 Intra-uterine system (Mirena) 11 Natural Family Planning

6 I have not used contraception previously

11. **Your Opinion**

For each contraceptive method listed below, please indicate whether you agree or disagree with the following statements, by circling the appropriate answer.

| **The daily Pill** | **Strongly agree** | **Agree** | **No opinion** | **Disagree** | **Strongly disagree** | **Do not know** |
| --- | --- | --- | --- | --- | --- | --- |
| The Pill prevents pregnancy effectively | 1 | 2 | 3 | 4 | 5 | 6 |
| The Pill has many side effects | 1 | 2 | 3 | 4 | 5 | 6 |
| Taking the Pill can be dangerous for your health | 1 | 2 | 3 | 4 | 5 | 6 |
| The Pill is easy to use | 1 | 2 | 3 | 4 | 5 | 6 |
| The Pill is easy to forget | 1 | 2 | 3 | 4 | 5 | 6 |
| The Pill gives you regular menstrual bleeding | 1 | 2 | 3 | 4 | 5 | 6 |
| The Pill protects against certain forms of cancer | 1 | 2 | 3 | 4 | 5 | 6 |
| Many women use the Pill | 1 | 2 | 3 | 4 | 5 | 6 |

| **The weekly patch** | **Strongly agree** | **Agree** | **No opinion** | **Disagree** | **Strongly disagree** | **Do not know** |  |
| --- | --- | --- | --- | --- | --- | --- | --- |
| The patch prevents pregnancy effectively | 1 | 2 | 3 | 4 | 5 | 6 |  |
| The patch has many side effects | 1 | 2 | 3 | 4 | 5 | 6 |  |
| Using the patch can be dangerous for your health | 1 | 2 | 3 | 4 | 5 | 6 |  |
| The patch is easy to use | 1 | 2 | 3 | 4 | 5 | 6 |  |
| Starting a new patch is easy to forget | 1 | 2 | 3 | 4 | 5 | 6 |  |
| The patch gives you regular menstrual bleeding | 1 | 2 | 3 | 4 | 5 | 6 |  |
| The patch protects against certain forms of cancer | 1 | 2 | 3 | 4 | 5 | 6 |  |
| Many women use the patch | 1 | 2 | 3 | 4 | 5 | 6 |  |

| **The monthly ring** | **Strongly agree** | **Agree** | **No opinion** | **Disagree** | **Strongly disagree** | **Do not know** |  |
| --- | --- | --- | --- | --- | --- | --- | --- |
| The ring prevents pregnancy effectively | 1 | 2 | 3 | 4 | 5 | 6 |  |
| The ring has many side effects | 1 | 2 | 3 | 4 | 5 | 6 |  |
| Using the ring can be dangerous for your health | 1 | 2 | 3 | 4 | 5 | 6 |  |
| The ring is easy to use | 1 | 2 | 3 | 4 | 5 | 6 |  |
| Starting a new ring is easy to forget | 1 | 2 | 3 | 4 | 5 | 6 |  |
| The ring gives you regular menstrual bleeding | 1 | 2 | 3 | 4 | 5 | 6 |  |
| The ring protects against certain forms of cancer | 1 | 2 | 3 | 4 | 5 | 6 |  |
| Many women use the ring | 1 | 2 | 3 | 4 | 5 | 6 |  |

**12. Please rate the information you received about contraception** (by circling the appropriate answer)**:**

|  | **Very** | **Somewhat** | **Neutral** | **Not very** | **Not at all** |
| --- | --- | --- | --- | --- | --- |
| Was it useful? | 1 | 2 | 3 | 4 | 5 |
| Was it complete? | 1 | 2 | 3 | 4 | 5 |
| Was it fair and balanced? | 1 | 2 | 3 | 4 | 5 |

**13. Which contraceptive method are you choosing, now that you have read the information materials and spoken to your doctor? (Please tick only one answer)**

1 Daily Pill 🡪 please go to question **14**

2 Weekly Patch 🡪 Please go to question **15**

3 Monthly Ring 🡪 Please go to question **16**

4 Other method 🡪 Please go to question **17**

5 Not yet decided 🡪 Please go to question **18**

**14. Only for women who chose the daily Pill**

**Please indicate the reasons why you selected the daily Pill.**

(Please select all that apply)

14a.

| Reasons to choose the **Daily Pill** | 0 Daily use  0 Will not forget it  0 Convenience  0 Easy to use  0 My friend uses it  0 I am used to it  0 Discrete  0 Recommended by my doctor | 0 Low hormone levels  0 Well-researched method  0 Regular menstrual bleeding  0 Low chance of side effects  0 Not dangerous  0 Relief from menstrual pain  0 Relief from acne  0 Other: __________________ |
| --- | --- | --- |

**Please indicate the reasons why you did NOT select the weekly patch or monthly ring.**

(Please select all that apply)

14b. & 14c.

| Reasons **NOT** to choose the Monthly Ring | 0 Not interested in monthly  contraception  0 More convenient methods  are available  0 Not easy to use  0 Heard negative stories  0 Not effective  0 Will forget to remove and replace  0 No regular menstrual bleeding  0 Cost | 0 Don’t like to use foreign body  0 Don’t know anybody who uses it  0 Doctor did not recommend it  0 Not comfortable inserting ring in  vagina  0 Can fall out  0 My partner does not like it  0 Side effects  0 Dangerous  0 Other: __________________ |
| --- | --- | --- |
| Reasons **NOT** to choose the Weekly Patch | 0 Not interested in weekly  contraception  0 More convenient methods  are available  0 Not easy to use  0 Heard negative stories  0 Not effective  0 Will forget to remove and replace  0 Don’t like to detach from skin  0 No regular menstrual bleeding  0 Cost | 0 Not discrete, visible  0 Can fall off  0 Can irritate skin  0 Don’t know anybody who uses it  0 Doctor did not recommend it  0 My partner does not like it  0 Side effects  0 Dangerous  0 Other: __________________ |

**This is the end of the questionnaire.**

**Thank you for your cooperation.**

**15. Only for women who chose the weekly Patch**

**Please indicate the reasons why you selected the weekly Patch.**

(Please select all that apply)

15a.

| Reasons to choose the **Weekly Patch** | 0 Weekly use  0 Will not forget it  0 Convenience  0 Easy to use  0 My friend uses it  0 I am used to it  0 Can check it, visible  0 Recommended by my doctor | 0 Low hormone levels  0 Still effective if I experience  vomiting or diarrhea  0 Regular menstrual bleeding  0 Low chance of side effects  0 Not dangerous  0 Relief from menstrual pain  0 Relief from acne  0 Other: __________________ |
| --- | --- | --- |

**Please indicate the reasons why you did NOT select the monthly ring or daily Pill.**

(Please select all that apply)

15b. & 15c.

| Reasons **NOT** to choose the Monthly Ring | 0 Not interested in monthly  contraception  0 More convenient methods  are available  0 Not easy to use  0 Heard negative stories  0 Not effective  0 Will forget to remove and replace  0 No regular menstrual bleeding  0 Cost | 0 Don’t like to use foreign body  0 Don’t know anybody who uses it  0 Doctor did not recommend it  0 Not comfortable inserting ring in  vagina  0 Can fall out  0 My partner does not like it  0 Side effects  0 Dangerous  0 Other: __________________ |
| --- | --- | --- |
| Reasons **NOT** to choose the Daily Pill | 0 Daily use  0 More convenient methods  are available  0 Not easy to use  0 Heard negative stories  0 Not effective  0 Will forget to take it  0 No regular menstrual bleeding  0 Very old method  0 Cost | 0 Not effective if I use certain  antibiotics  0 Efficacy reduced by vomiting,  diarrhea  0 Don’t know anybody who uses it  0 Doctor did not recommend it  0 My partner does not like it  0 Side effects  0 Dangerous  0 Other:_________________ |

**This is the end of the questionnaire.**

**Thank you for your cooperation.**

**16. Only for women who chose the monthly Ring**

**Please indicate the reasons why you selected the monthly Ring.**

16a.

(Please select all that apply)

| Reasons to choose the **Monthly Ring** | 0 Monthly use  0 Will not forget it  0 Convenience  0 Easy to use  0 My friend uses it  0 I am used to it  0 Discretion  0 Recommended by my doctor | 0 Steady, low hormone levels  0 Still effective if I use certain  antibiotics  0 Still effective if I experience  vomiting, diarrhea  0 Regular menstrual bleeding  0 Low chance of side effects  0 Not dangerous  0 Relief from menstrual pain  0 Relief from acne  0 Other: _________________ |
| --- | --- | --- |

**Please indicate the reasons why you did NOT select the daily Pill or weekly patch.**

(Please select all that apply)

16b. & 16c.

| Reasons **NOT** to choose the Daily Pill | 0 Daily use  0 More convenient methods  are available  0 Not easy to use  0 Heard negative stories  0 Not effective  0 Will forget to take it  0 No regular menstrual bleeding  0 Very old method  0 Cost | 0 Not effective if I use certain  antibiotics  0 Efficacy reduced by vomiting,  diarrhea  0 Don’t know anybody who uses it  0 Doctor did not recommend it  0 My partner does not like it  0 Side effects  0 Dangerous  0 Other:_________________ |
| --- | --- | --- |
| Reasons **NOT** to choose the Weekly Patch | 0 Not interested in weekly  contraception  0 More convenient methods  are available  0 Not easy to use  0 Heard negative stories  0 Not effective  0 Will forget to remove and replace  0 Don’t like to detach from skin  0 No regular menstrual bleeding  0 Cost | 0 Not discrete, visible  0 Can fall off  0 Can irritate skin  0 Don’t know anybody who uses it  0 Doctor did not recommend it  0 My partner does not like it  0 Side effects  0 Dangerous  0 Other: __________________ |

**This is the end of the questionnaire.**

**Thank you for your cooperation.**

1. **Only for women who chose another method**

**17a.**

**Which method of contraception have you chosen?**

**1** Estrogen-free / Progestogen-only Contraceptive Pill

2 Intra-uterine system (Mirena)

3 Intra-uterine device (other than Mirena)

4 Contraceptive implant (Implanon or like)

5 Contraceptive injection (Depo-Provera or like)

6 Sterilization

7 Condoms

8 Other, ____________________________________________

**17b.**

**Please indicate the reasons why you selected this method.**

(Please select all that apply)

| Reasons to choose **your** **method** | 0 Very effective method  0 Will not forget it  0 Convenience  0 Easy to use  0 My friend uses it  0 I am used to it  0 Discrete  0 Recommended by my doctor  0 Long-acting method  0 Don’t need doctor prescription | 0 Family is complete  0 Low chance of side effects  0 Not dangerous  0 Cannot use other methods  0 Heard positive stories about it  0 My partner recommended it  0 My partner wants to take his  Responsibility  0 Relief from menstrual pain  0 Other: __________________ |
| --- | --- | --- |

**See next page for continuing question.**

**17c, 17d & 17e**

**Please indicate the reasons why you did NOT select the daily Pill, the weekly patch or the monthly ring.**

(Please select all that apply)

| Reasons **NOT** to choose the Daily Pill | 0 Daily use  0 More convenient methods  are available  0 Not easy to use  0 Heard negative stories  0 Not effective  0 Will forget to take it  0 No regular menstrual bleeding  0 Very old method  0 Cost | 0 Not effective if I use certain  antibiotics  0 Efficacy reduced by vomiting,  diarrhea  0 Don’t know anybody who uses it  0 Doctor did not recommend it  0 My partner does not like it  0 Side effects  0 Dangerous  0 Other:_________________ |
| --- | --- | --- |
| Reasons **NOT** to choose the Monthly Ring | 0 Not interested in monthly  contraception  0 More convenient methods  are available  0 Not easy to use  0 Heard negative stories  0 Not effective  0 Will forget to remove and replace  0 No regular menstrual bleeding  0 Cost | 0 Don’t like to use foreign body  0 Don’t know anybody who uses it  0 Doctor did not recommend it  0 Not comfortable inserting ring in  vagina  0 Can fall out  0 My partner does not like it  0 Side effects  0 Dangerous  0 Other: __________________ |
| Reasons **NOT** to choose the Weekly Patch | 0 Not interested in weekly  contraception  0 More convenient methods  are available  0 Not easy to use  0 Heard negative stories  0 Not effective  0 Will forget to remove and replace  0 Don’t like to detach from skin  0 No regular menstrual bleeding  0 Cost | 0 Not discrete, visible  0 Can fall off  0 Can irritate skin  0 Don’t know anybody who uses it  0 Doctor did not recommend it  0 My partner does not like it  0 Side effects  0 Dangerous  0 Other: __________________ |

**This is the end of the questionnaire.**

**Thank you for your cooperation.**

**18. Only for women who have not yet chosen their method**

**Why have you not yet chosen a contraceptive method?**

1 Unanswered questions

2 Want to discuss with partner

3 Want to discuss with others

4 Want to get further information

5 No immediate need for contraception

6 Other reason _____________________________ (please complete)

**This is the end of the questionnaire.**

**Thank you for your cooperation.**
