# Supplementary material for: Observational, prospective, multicentre study to evaluate the effects of counselling on the choice of combined hormonal contraceptives in Italy—the ECOS (Educational COunselling effectS) study
Source: BMC Womens Health. 2015 Sep 2;15:69. doi: 10.1186/s12905-015-0226-x (PMC4557636; doi:10.1186/s12905-015-0226-x)
Supplement: Additional file 3: — Leaflet. The file provides the counselling leaflet distributed to the subjects. (PDF 2810 kb) [file 12905_2015_226_MOESM3_ESM.pdf]

TF03001_ Con_Coun_Tool_final_rev4:Layout 1 3/13/09 10:37 AM Page 1

Combination Contraceptive Methods

All of these methods contain a fixed combination of estrogen and progesterone. The main differences lie in the frequency of use and the route of administration, which may represent important differences in terms of convenience.

**Method**	**Daily Pill** 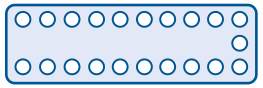	**Weekly Transdermal Patch** 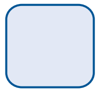	**Monthly Ring** 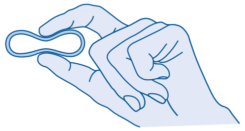
**Description**	A package of 21 pills with estrogen and progestogen. Each pill is used for one day of contraception.	Three adhesive skin patches with estrogen and progestogen. Each patch is used for one week of contraception.	One flexible, vaginal, plastic ring with estrogen and progestogen. The ring is used for one month of contraception.
**Effectiveness (when used as directed)**	99%	99%	99%
**Return to Fertility**	Fertility usually returns quickly once contraception method is stopped.		
**Specific Characteristics**	• Most studied method, with more experience than other methods<br>• Regular menstrual bleeding patterns<br>• Effectiveness depends on daily administration<br>• The pill releases the hormones into the digestive tract<br>– Efficacy affected by vomiting and diarrhea<br>• Daily fluctuation in hormone levels<br>• Can be used to delay menstruation, if desired<br>• Some noncontraceptive benefits proven	• Regular menstrual bleeding patterns<br>• No need to remember everyday<br>• Greater incidence of breast tenderness<br>• Not recommended for women weighing more than 90 Kg<br>• Estrogen levels (over 21 days) higher than with pill or ring*<br>• Can be used to delay menstruation, if desired<br>• The patch releases the hormones across the skin<br>– Greater incidence of skin irritation and allergic skin reactions<br>• Noncontraceptive benefits likely to be present but not proven	• Very regular and predictable menstrual bleeding patterns<br>• No need to remember everyday<br>• The ring releases the hormones across the vaginal wall<br>– Greater incidence of vaginal or device-related symptoms<br>• Minimal awareness of the ring during sexual relations and everyday life<br>• Continuous low-dose estrogen levels (over 21 days)*<br>• Can be used to delay menstruation, if desired<br>• Noncontraceptive benefits likely to be present but not proven
**Mode of Administration**	Oral	Transdermal	Vaginal
**Use While Breastfeeding**	Should not be used while breastfeeding.		
**STD and HIV protection**	Does not protect against sexually transmitted diseases and HIV.		
**Possible side effects**	Irregular menstrual bleeding, headache, vomiting, diarrhea, abdominal pain, weight increase (fluid retention), depressed mood, mood altered, breast pain		

For some women, progestogen-only methods (minipill, injection, implant, or intrauterine system) may be a good alternative.
These methods are highly effective and can be used while breastfeeding. The main side effect is unpredictable menstrual bleeding.

*Amount of estrogen exposure (ethinyl estradiol: EE) in the blood over 21 days. The clinical significance of these findings, if any, is unknown.

Developed in consultation with the European Society of Contraception and Reproductive Health.

Choice
Contraceptive Health Research
Of Informed Choice Experience
